# Identification and Screening of Novel Antioxidant Peptides from Yak Skin and Their Protective Effect on H_2_O_2_-Induced HepG2 Cells Oxidation

**DOI:** 10.3390/ijms26135976

**Published:** 2025-06-21

**Authors:** Yan Jin, Nan Zhang, Yurong Huang, Ziyao Zhang, Enhui Jin, Yu Kong, Wenjie Sui, Tao Wu, Min Zhang

**Affiliations:** 1 College of Food Science and Engineering, Tianjin University of Science & Technology, Tianjin 300457, China; jinyan@tust.edu.cn (Y.J.); nzhanga@126.com (N.Z.); hyrong0420@163.com (Y.H.); ziyaozhang123@163.com (Z.Z.); 15641660860@163.com (E.J.); yu_kong@tust.edu.cn (Y.K.); wjsui@tust.edu.cn (W.S.); 2 College of Food Science and Bioengineering, Tianjin Agricultural University, Tianjin 300384, China

**Keywords:** yak skin, antioxidant peptide, molecular docking, oxidative stress, Keap1-Nrf2/ARE pathway

## Abstract

To improve the bioavailability of yak by-products, novel antioxidant peptides were prepared and identified from yak skin hydrolysate. The results showed that the ultrafiltration fraction of a molecular weight of less than 1 kDa had the strongest free radical scavenging activity. A total of 219 novel peptides were identified by mass spectrometry and five antioxidant peptides were screened based on molecular docking with Keap1 (LMGPR, GFDGD, FGFDGDF, GHNGLDGL, and GPAGPQGPR). These peptides may bind with Keap1 competitively and exert antioxidant effects by activating the Nrf2/ARE pathway. After synthesis, FGFDGDF showed a better free radical scavenging ability and protective effect on H_2_O_2_-induced oxidative damage of HepG2 cells among these peptides. The pretreatment of peptides could enhance the activity of intracellular antioxidant enzymes and reduce the level of malondialdehyde and IL-8. This study provides a scientific basis for the application of yak skin peptide as a novel antioxidant in functional food.

## 1. Introduction

Free radicals are inevitable products of metabolism and are usually eliminated by the antioxidant defense system, which is an ongoing balancing process. When stimulated by external factors, this balance might be disrupted, leading to an excessive production of free radicals and initiating oxidative stress to damage health [1]. Although synthetic antioxidants such as butylated hydroxyanisole, tert-butylhydroquinone, and propyl gallate have the ability to reduce free radicals, their potential toxicity and carcinogenicity limit their use in food application [2]. Therefore, the development of natural and non-toxic antioxidants has emerged as a key research focus. In recent years, an increasing number of antioxidant peptides have been identified and derived primarily from legumes, animal-based foods, and marine proteins, which were typically released from proteins through enzymatic hydrolysis [3,4,5]. Protease hydrolysis is the preferred technique for producing antioxidant peptides due to its high selectivity, mild reaction conditions, minimal side reactions and high efficiency selectivity [6]. Studies have shown that the antioxidant activity of peptides is affected by molecular weight, the type of amino acid, and their sequence. It was found that the molecular weight of the optimal antioxidant activity of rice, walnut, and other protein hydrolysates prepared by enzymatic hydrolysis was concentrated below 1 kDa [7,8]. Overall, a reasonable combination of molecular weight and amino acid sequence is crucial for enhancing the activity of antioxidant peptides. Therefore, the extensive use of different food resources and high-efficiency preparation progress on antioxidant peptides with strong antioxidant activity and stability still need to be investigated.

Yak (*Bos grunniens*) is a rare breed mainly found in the Qinghai–Tibet Plateau. China harbors the largest population and diversity of yaks accounting for more than 94% worldwide [9]. Because of the harsh environment of extreme coldness, hypoxia, and strong radiation, yak products may have unique properties regarding nutrition, sensory characteristics, and processing properties [10]. As the main by-product of yak processing in meat and milk, yak skin (YS) is rich in protein, accounting for 30–40%, which can be a good resource of bioactive peptides [11]. Nowadays, YS is usually discarded or is traditionally utilized in low-quality leather production, leading to a wastage of this valuable resource. Studies show that YS gelatin has great potential to replace commercial gelatin because of its superior properties including oxidation resistance, emulsification, and heat resistance [12]. Its collagen has whitening, moisturizing, and wrinkle-eliminating effects, and is easy to absorb, with good biocompatibility and degradability. Xiaotong Ma et al. [13] identified three novel antifreeze peptides from YS hydrolysates which could increase the survival rate of probiotics. Moreover, YS boasts outstanding nutritional values, which may apply in the mitigation of myocardial ischemia–reperfusion injury [14]. The potential angiotensin I-converting enzyme (ACE)-inhibitory activities of YS gelatin peptides were also investigated, but the sequences were not clarified [15]. Only two novel peptides (GADGAPGKDGVRG and GPRGDQGPVGR) with a good ability for iron-chelating were identified and studied from YS [16]. Overall, current research mainly focuses on gelatin, while the development of bioactive peptides derived from YS is limited. Further research on the preparation, identification, and healthy functions of yak skin peptides (YSP) needs to be clarified, as well as the specific mechanism of its bioactivity.

The molecular docking techniques based on machine learning and artificial intelligence are widely used in the area of functional food and drug development [17]. Molecular docking is derived from Fisher E’s lock and key model, which posits that the spatial alignment of receptors and ligands is crucial for their recognition [18]. The interaction between peptides and biological targets can be directly observed through molecular docking to elucidate the biological mechanism of food-derived peptides [19]. Nuclear factor erythrocyte-associated factor 2 (Nrf2) is recognized as a neurotherapeutic target, and the Keap1-Nrf2/ARE pathway serves as a pivotal signaling pathway for endogenous oxidant damage [20]. In recent years, research has focused on exploring the mechanism of antioxidant peptides through Keap1-Nrf2/ARE pathway [21], such as the milk-derived peptide KVLPVPEK (K-8-K), which inhibits lipid peroxidation and prevents the interaction between Nrf2 and Keap1 [22]. These comprehensive methods offer robust support for the research and application of antioxidant peptides.

Therefore, in this study, antioxidant peptides were prepared from YS through enzymatic hydrolysis and ultrafiltration separation, identified by mass spectrometry and screened based on molecular docking with Keap1. Then the cell protection effect of YSP was determined by using the H_2_O_2_-induced oxidative damage model of HepG2 cells. This study provides a theoretical foundation for the development and application of YSP, aiming to improve the high-value utilization of YS and broaden the source of antioxidants.

## 2. Results and Discussion

### 2.1. Structural Characterization of YSP

**Figure 1:**
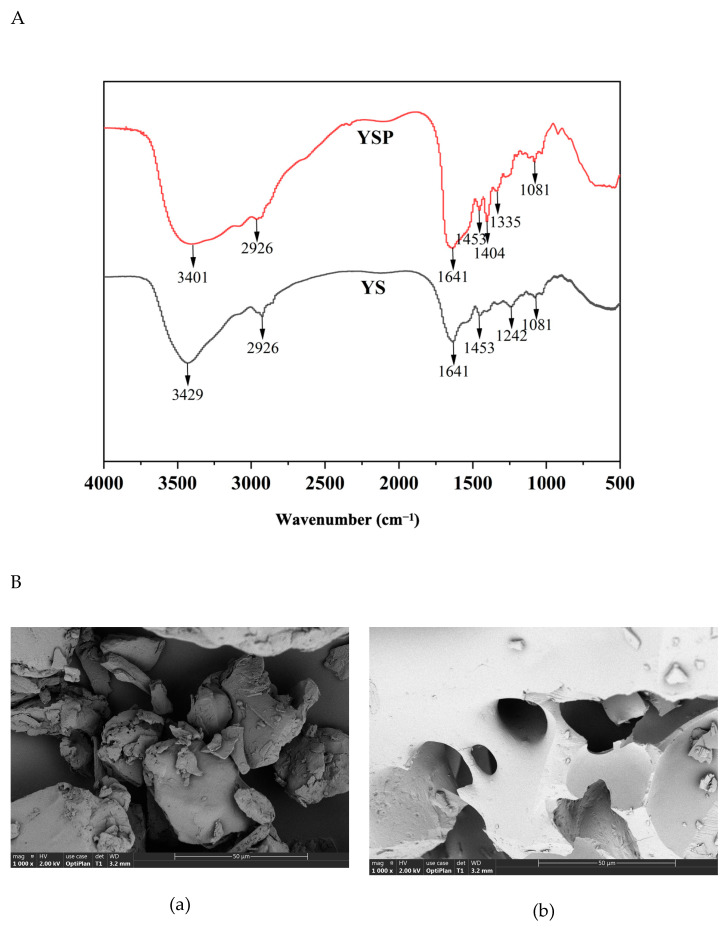
Fourier transform infrared spectroscopy of YS and YSP (**A**) and the SEM images of YS (**B**-**a**) and YSP (**B**-**b**) (1000×).

The structural characterization on YS and YSP are shown in Figure 1A. Both YS and YSP had a special absorption peak in the range of 3500–3300 cm^−1^, which was mainly associated with N-H and O-H stretching. It reflected the presence of hydrogen bonds in the peptide chain skeleton [23]. Both samples exhibited an absorption peak at 2926 cm^−1^, which was caused by asymmetric C-H stretching vibration, indicating that both samples contained aliphatic amino acids side chain [24]. In addition, absorption was observed at 1641 cm^−1^ in the range of amide I band (1600–1700 cm^−1^), indicating that the protein had a more complete triple helix structure. Moreover, the absorption peak at 1453 cm^−1^ and 1081 cm^−1^ also appeared in both YS and YSP. It indicated that the secondary structures of both samples were relatively stable. Notably, YSP showed several different groups compared with YS, including C=O bond vibration in carboxylic acid (1404 cm^−1^) and C-H bond stretching vibration (1335 cm^−1^). These differences indicated that the protein structure was unfolded, and its secondary structure might be destroyed by enzymatic hydrolysis. These changes might expose the originally tightly wrapped active groups, making the enzyme hydrolysis product more likely to capture electrons, thereby exhibiting improved antioxidant capacity.

In addition, the microstructure of YS and YSP had great differences, as shown in Figure 1B. YS was observed to be a dense block with an irregular concave surface. After enzymatic hydrolysis, YSP exhibited a large number of porous and fracture microstructures with a relatively loose texture. This may be due to the enzymatic hydrolysis-induced destruction of hydrogen bonds and van der Waals forces between protein molecules, which weaken the intermolecular interaction, resulting in a certain degree of stretching of protein molecules [25]. This result was consistent with previous reports indicating that proteins were broken down into small fragments, and they adopt a loosely structured conformation after enzyme treatment [26,27]. These structural changes might result in more hydrophobic amino acids being exposed to the surface, which could enhance the antioxidant capacity of the sample.

### 2.2. Free Radical Scavenging Activities of Ultrafiltrate Fractions

**Figure 2:**
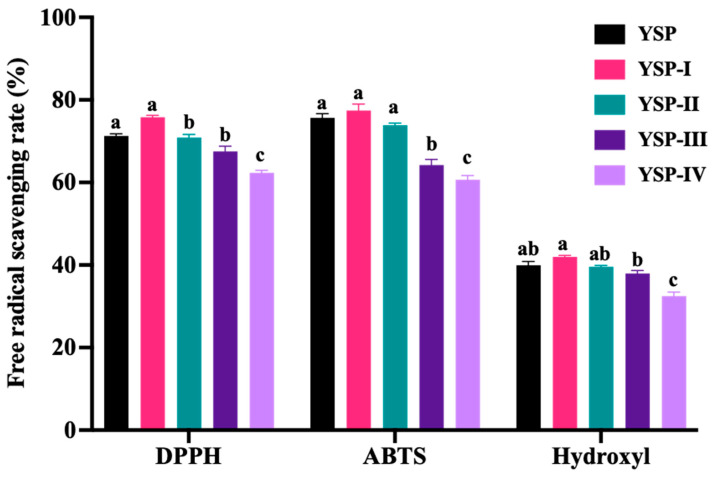
Free radical scavenging activity of YSP and ultrafiltration fractions, including YSP-I (<1 kDa), YSP-II (1–3 kDa), YSP-III (3–10 kDa), and YSP-IV (>1 kDa). Different letters represent significant differences (*p* < 0.05).

To accurately screen and identify its components, YSP was separated by ultrafiltration membranes. The free radical scavenging activities of four fractions are shown in Figure 2. Obviously, the components with a molecular weight of less than 1 kDa (YSP-I) exhibited the highest scavenging rate on the DPPH radical (75.80 ± 0.76%), ABTS radical (77.42 ± 2.77%), and hydroxyl radical (41.96 ± 0.67%) compared to other fractions, with no significant difference from YSP. It indicated that the components with lower molecular weight might have a stronger antioxidant ability. Similarly, Yawen Kong et al. [28] reported that the molecular weight of less than 3 kDa demonstrated excellent antioxidant activity. Long He et al. [29] reported that the radical scavenging ability of DPPH and the ABTS scavenging activity of yak skin gelatin hydrolysates were significantly increased after ultrasonic treatment. Overall, the components with a molecular weight of <1 kDa were selected for further analysis.

### 2.3. Identification and Screening of Antioxidant Peptide from YSP by LC-MS/MS

**Figure 3:**
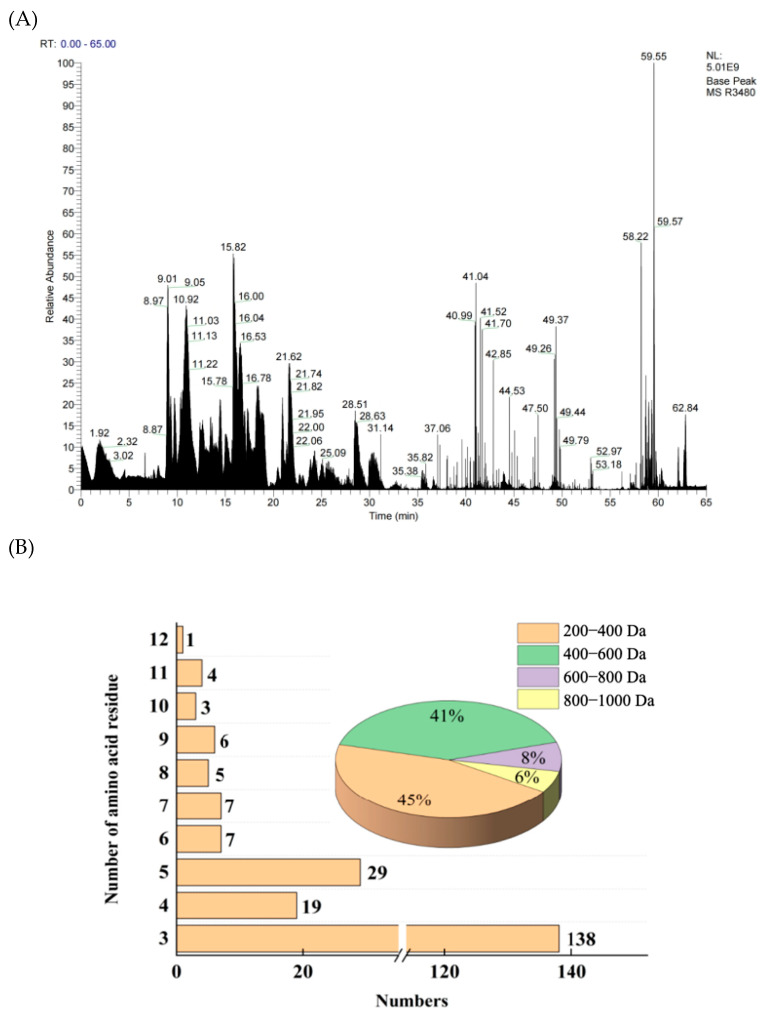
Total ion diagram of mass spectrum (**A**) and the peptides distribution (**B**) by LC-MS/MS.

YSP-I samples were further analyzed by LC-MC/MC, and 219 peptides were identified (Supplementary Table S1). This showed that YSP-I was mainly composed of short peptides, where 138 peptides were tripeptides and the molecular weight of peptides were mainly concentrated between 200 and 600 Da, accounting for 86% (Figure 3). It indicated that YS protein was effectively hydrolyzed by trypsin. Furthermore, an online bioinformatics analysis was performed to predict the potential biological activities of these peptides. The peptides with a probability threshold where the peptide ranker exceeded 0.5 were selected for further analysis [30]. Meanwhile, non-toxicity, non-hypersusceptibility, and good water solubility were the key factors for screening highly specific peptides [31,32,33]. The good solubility of small molecular weight peptides could react with lipid free radicals, and they had strong antioxidant activity [4]. Based on these, 29 peptides with potential antioxidant activity, good water solubility, and non-toxicity were selected as shown in Table 1. The molecular weight of these peptides ranged from 259.11682 to 950.40937 Da, and the number of amino acid residues ranged from 3 to 11. Meanwhile, the 29 novel peptides have not been reported before.

### 2.4. Molecular Docking Simulation of Antioxidant Peptides Interacting with Keap1

The Keap1-Nrf2 signaling pathway was recognized as an important mechanism, in which bioactive compounds exerted antioxidant effects [34]. When oxidative damage occurred, excessive free radicals interacted with Keap1, and then Nrf2 was released and up-regulated the antioxidant response element (ARE) to reduce oxidative damage [20]. A large number of studies have shown that peptides might change the Keap1/Nrf2-ARE signaling pathway and thereby affect the activity of antioxidant enzymes in cells [35]. In this study, YSP has exhibited good free radical scavenging activity, but its molecular mechanisms remained unclear. Therefore, the molecular docking analysis was conducted to explore the molecular mechanism of antioxidant peptides by interacting with Keap1.

**Figure 4:**
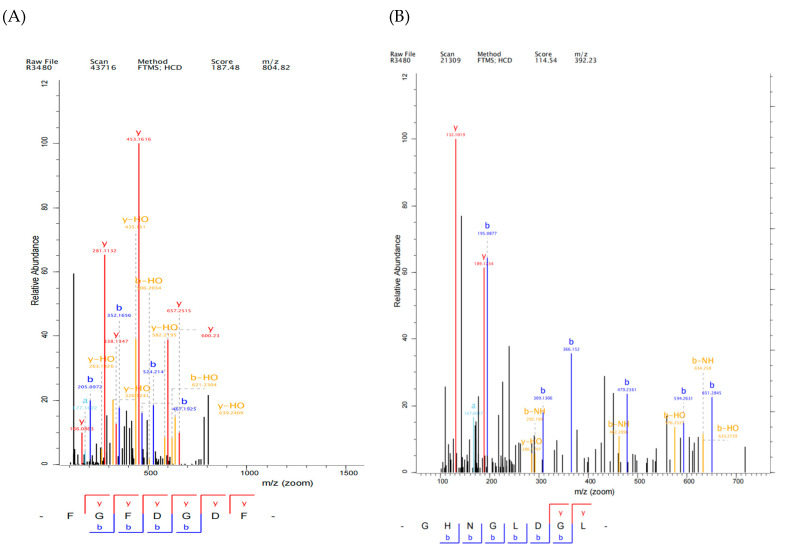

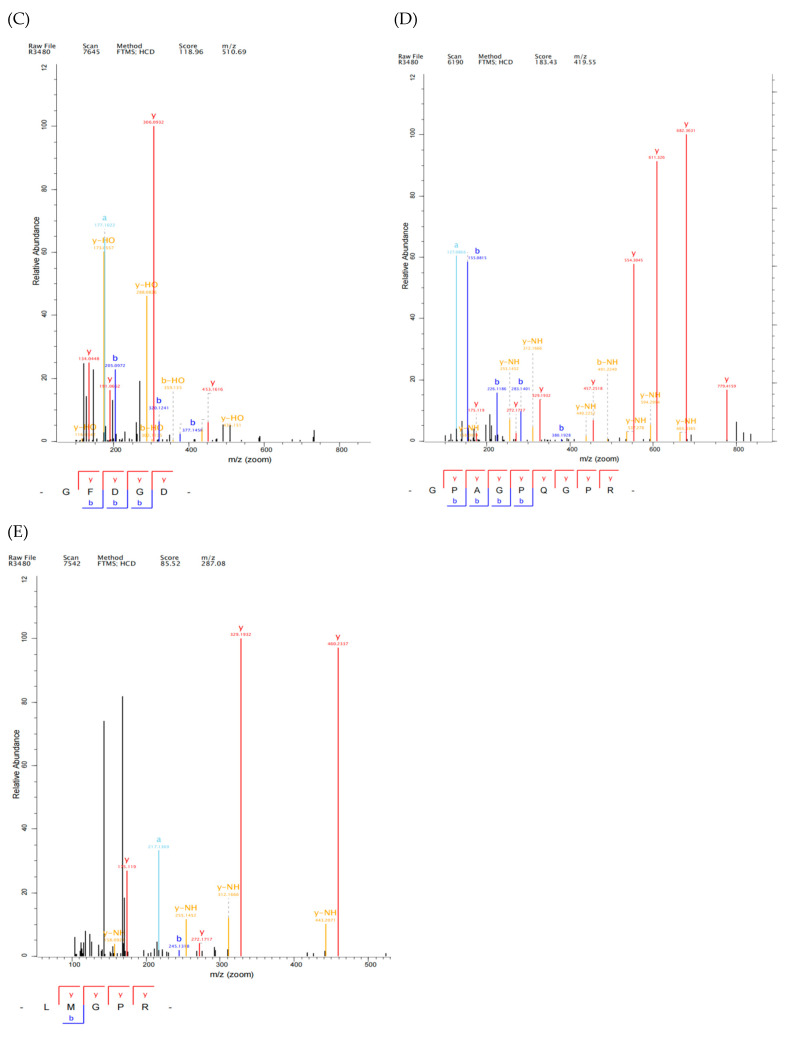
Secondary mass spectrum of five peptides, FGFDGDF (**A**), GHNGLDGL (**B**), GFDGD (**C**), GPAGPQGPR (**D**), and LMGPR (**E**).

It was found that peptides with lower binding energy exhibited stronger binding affinity with Keap1 protein [36]. In the present study, the binding energy values of 29 peptides were ranked (Table 1) and five novel peptides were selected, including Phe-Gly-Phe-Asp-Gly-Asp-Phe (FGFDGDF), Gly-His-Asn-Gly-Leu-Asp-Gly-Leu (GHNGLDGL), Gly-Phe-Asp-Gly-Asp (GFDGD), Gly-Pro-Ala-Gly Pro-Gln-Gly-Pro-Arg (GPAGPQGPR), Leu-Met-Gly-Pro-Arg(LMGPR) (secondary mass spectrum shown in Figure 4), where the CDocker energy binding with Keap1 were −141.445 kcal/mol, −136.723 kcal/mol, −115.582 kcal/mol, −83.766 kcal/mol and −68.852 kcal/mol, respectively. FGFDGDF showed the strongest affinity with Keap1 protein. Studies showed that hydrophobic amino acids (such as Leu, Val, Ala, Pro, and Phe), aromatic amino acids, and basic amino acids contributed to the antioxidant activity of peptides [37]. Hydrophobic amino acids at the N-terminal or C-terminal could enhance the interaction of peptides with lipid components to promote their antioxidant function in vivo [38]. Pro, Ala, and Tyr were the key amino acids for free radical scavenging, while His exhibited strong antioxidant activity due to its imidazole group structure with a closed π bond arrangement and unpaired electrons [39]. These amino acids existed in the five screened peptides; it can be inferred that these peptides may possess good antioxidant activities.

**Figure 5:**
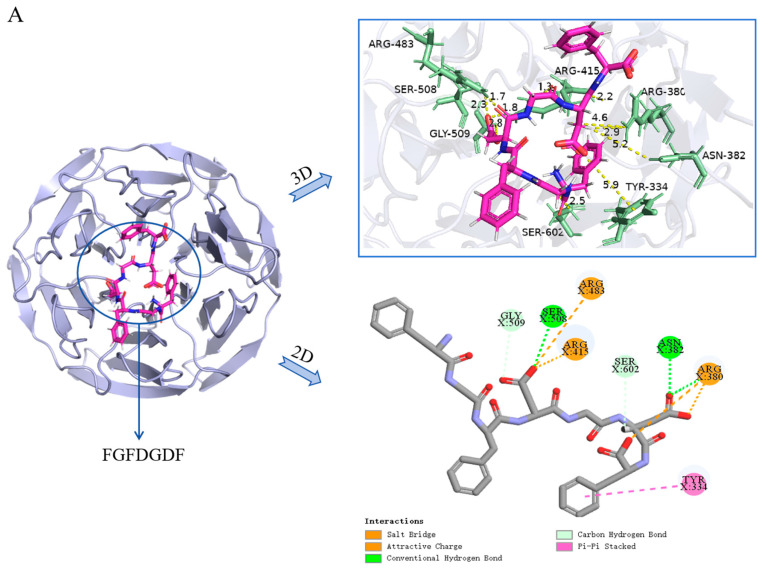

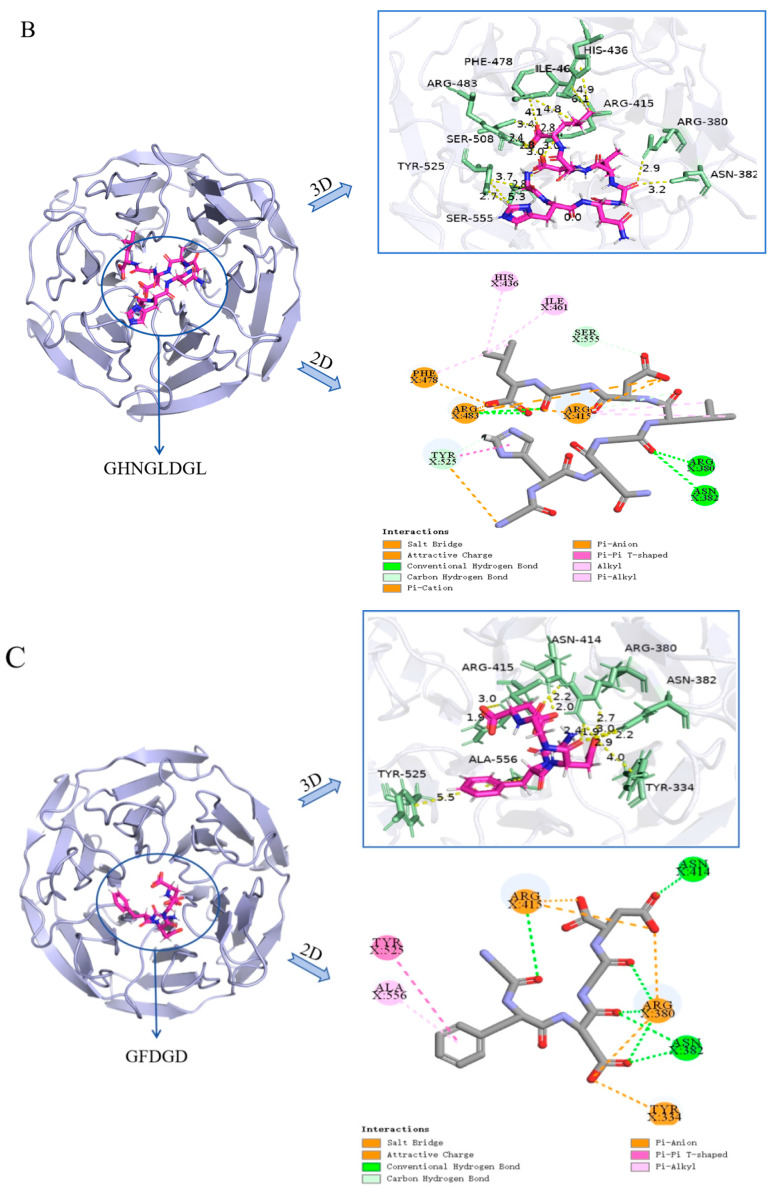

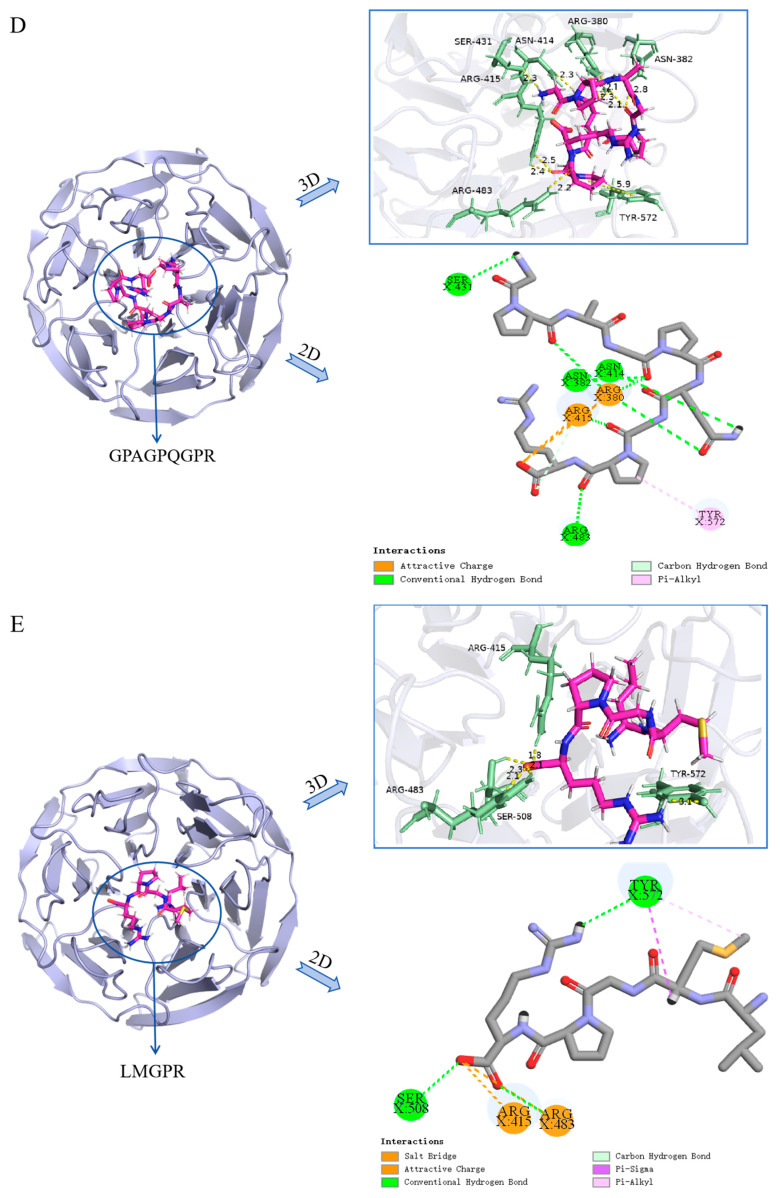
The 2D and 3D simulation diagrams of five antioxidant peptides binding with Keap1 (PDB ID:2FLU) by molecular docking, FGFDGDF (**A**), GHNGLDGL (**B**), GFDGD (**C**), GPAGPQGPR (**D**), and LMGPR (**E**), respectively.

According to the crystal structure of the Keap1-Nrf2 complex, the binding pocket can be divided into six bags, namely P1-P6 [40]. The interaction sites of peptides binding with Keap1 are shown in Figure 5. GFDGD was a part of FGFDGDF, and they shared the same active sites to bind with Keap1 (Figure 5A,C), including ASN-382, ARG-380, and ARG-415, which was basically consistent with the previous study [41]. Both FGFDGDF and GFDGD were bound to the P1, P2, P3, and P5 pockets of Keap1. Additionally, the GFDGD peptide was bound to the P4 and P6 active pockets because of its shorter chain. Considering the strong binding energy of FGFDGDF, further research is needed to in order to clarify whether adding or removing a specific amino acid could improve the antioxidant activity of the small peptide. As for the other three peptides, LMGPR was bound to the TYR-572 and ARG-483 residues in the P1 and P4 pockets of the Keap1 protein; GHNGLDGL was bound at the ARG-380, ASN-382 in P1-P4 and P6 pockets; and GPAGPQGPR interacted with SER-431, ASN-414, and other residues in the P1, P2, P4, and P6 pockets to form varieties of interactions. Keap1 mainly recognizes Nrf2 through ETGE and DLG motifs, which mainly binds to ARG-380, ARG-483, and other sites [42], which overlaps with the binding sites of identified peptides. Meanwhile, intermolecular hydrogen bonds, electrostatic interactions, and hydrophobic interactions were the main interactions observed between the five peptides and Keap1, which were previously reported [43]. These results showed that the five peptides could form novel and stable complexes with Keap1. In a word, all five peptides interacted with the six active pockets where Keap1 interacted with ligand Nrf2, and they were in contact with the key residues of Keap1-Nrf2 interactions. The results suggested that these peptides might induce Nrf2 release by competitively binding to Keap1, and they may exert antioxidant activity through activating the Keap1-Nrf2/ARE pathway. These peptides potentially facilitate the release of Nrf2 by competitively binding to Keap1. Subsequently, the activated Nrf2 is translocated into the nucleus, where it forms a heterodimer that binds to the antioxidant response element (ARE), thereby initiating the expression of target genes and exerting antioxidative effects [44]. Pei et al. [45]. also found that antioxidant peptides CRPCGPTP and ANSCNEPCVR, derived from feather keratin, could bind well to key amino acids in the Keap1 Kelch domain and directly disrupt the Keap1-Nrf2 interaction.

### 2.5. Free Radical Scavenging Activities of Peptides

**Figure 6:**
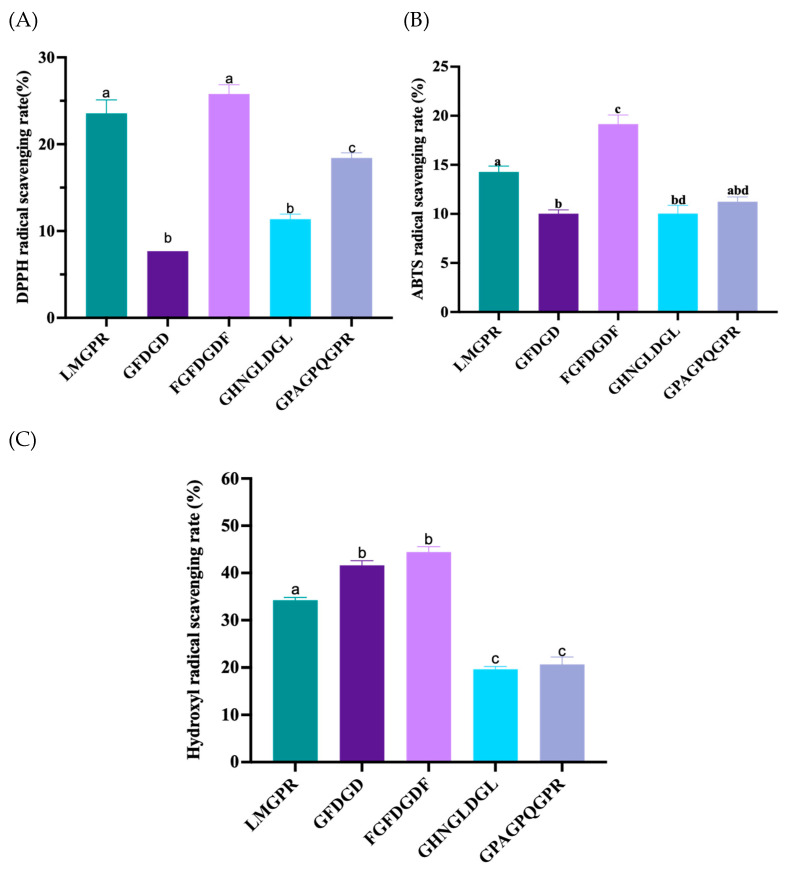
The free radical scavenging activities of synthetic peptides, including the DPPH (**A**), ABTS (**B**), and hydroxyl radical (**C**) scavenging rate of five peptides, respectively. Different letters represent significant differences (*p* < 0.05).

The above five screened peptides were synthesized, and their free radical scavenging activities are shown in Figure 6. FGFDGDF and LMGPR showed relatively better DPPH radical scavenging activities compared to the other three peptides, with significant differences (*p* < 0.05). The scavenging rates were 25.80% ± 1.82% and 23.56% ± 2.68%, respectively. FGFDGDF also demonstrated a better ABTS free radical scavenging activity than the other four peptides, with a significant difference (*p* < 0.05), and its clearance rate was 19.15% ± 1.59%. FGFDGDF and GFDGD showed higher hydroxyl radical scavenging activity than the remaining three peptides, with significant differences (*p* < 0.05), and their clearance rates were 44.44% ± 1.94% and 41.61% ± 1.73%, respectively. It can be observed that all five peptides reflected scavenging activity on the free radical. Among them, FGFDGDF had the best free radical scavenging activity, which was coincident with the results of molecular docking. However, the antioxidant activity of peptides may be limited due to the insufficient density of amino acid groups containing phenolic hydroxyl/indole groups such as tyrosine (Tyr) and tryptophan (Trp). Liu et al. [46] believed that the phenolic hydroxyl group of Tyr in the peptide chain was active. The antioxidant activity increased when Tyr was between DD (Asp-Asp) or DQ (Gln-Asp).

### 2.6. Protective Effect of Peptides on the Oxidative Damage of HepG2 Cells Induced by H_2_O_2_

**Figure 7:**
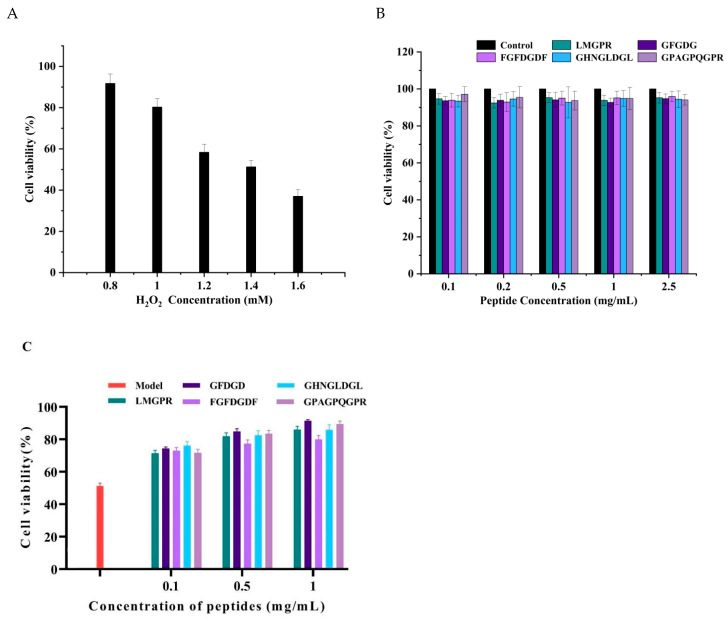
Effect of different concentrations of H_2_O_2_ (**A**) and five peptides (**B**) on the cell viability of HepG2 cells and the protective effect of peptides against oxidative stress induced by H_2_O_2_ (**C**)_._

To further verify the biological antioxidant activities of peptides, the protective effect on the oxidative damage of HepG2 cells was investigated. As a hepatotoxic chemical with a long half-life, H_2_O_2_ can be converted into hydroxyl free radicals and oxygen free radicals, directly causing the oxidative stress damage of cells [47]. As shown in Figure 7A, with the increase of H_2_O_2_ concentration from 0.8 µM to 1.6 mM, the cell viability significantly decreased from 91.77 ± 4.53% to 37.01 ± 3.20% (*p* < 0.05). Under the treatment with 1.4 mM H_2_O_2_, the cell survival rate was 51.29 ± 3.06%, which was selected to establish the cell oxidative damage model for subsequent experiments. Meanwhile, the cytotoxicity of peptides was also determined (Figure 7B). With the peptides concentration ranging from 0.1 mg/mL to 2.5 mg/mL, the cell viability was over 90% for all, which indicated that the five peptides had no toxic effect on the normal proliferation of HepG2 cells. When pretreated with antioxidant peptides, the cellular oxidative damage by H_2_O_2_ was reduced (Figure 7C). Compared with the model group, the survival rate of HepG2 cells significantly increased with the pretreatment concentration of five peptides. It indicated that the five peptides had a good protective effect on the oxidative damage of HepG2 cells.

### 2.7. Effects of Antioxidant Peptides on Antioxidant Enzymes, Malondialdehyde (MDA), and IL-8 Contents in H_2_O_2_-Induced HepG2 Cells

**Figure 8:**
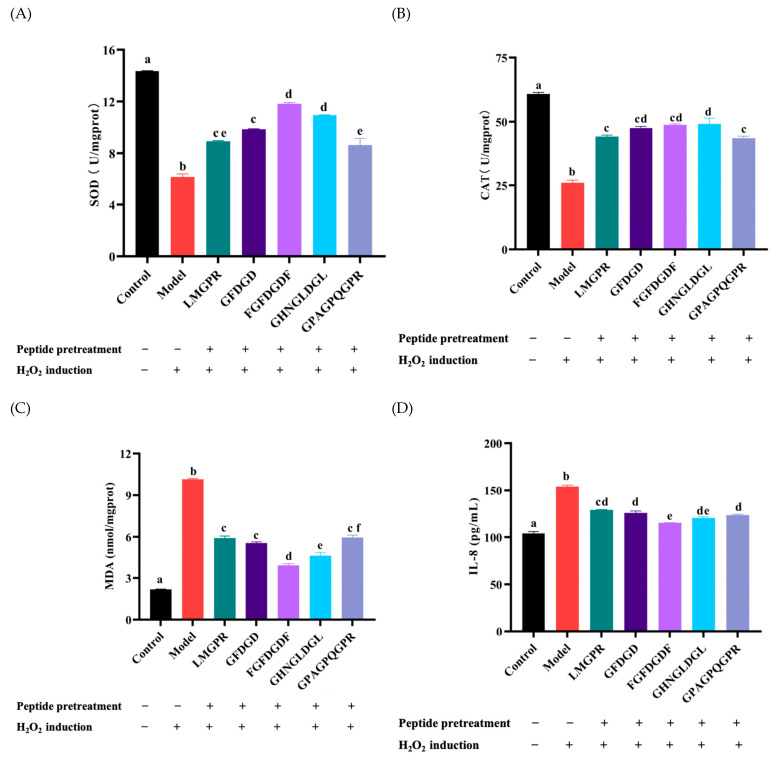
Effects of different peptides on the content of CAT (**A**), SOD (**B**), MDA (**C**), and IL−8 (**D**) in H_2_O_2_−induced HepG2 cells. Different letters represent significant differences (*p* < 0.05).

As shown in Figure 8A,B, the activities of CAT and SOD in HepG2 cells decreased to 26.01 ± 1.78 U/mg prot and 6.17 ± 0.41 U/mg pro, which were significantly lower than those in the control group (60.80 ± 1.10 U/mg prot, 14.35 ± 0.06 U/mg prot). Under the pretreatment of peptides (LMGPR, GFDGD, FGFDGDF, GHNGLDGL, GPAGPQGPR), the activities of SOD and CAT were significantly more improved than those in the model group (*p* < 0.05). The regulation of endogenous antioxidant enzymes SOD and CAT in cells is one of the main mechanisms of antioxidant peptides [48]. The results indicated that LMGPR, GFDGD, FGFDGDF, GHNGLDGL, and GPAGPQGPR could regulate the activity of antioxidant enzymes and protect the cells from oxidation damage. Specifically, FGFDGDF exhibited the best protective effect, where the activities of SOD and CAT in cells improved, ebing 11.83 ± 0.16 U/mg prot and 47.49 ± 1.14 U/mg prot, which were consistent with the above results.

Moreover, MDA is one of the key products of cell membrane lipid peroxidation under oxidative stress [49]. Studies have shown that the higher the MDA content, the more serious the cell damage [28]. As shown in Figure 8C, compared with the control group, the MDA content in HepG2 cells of the group significantly increased (10.31 ± 0.23 nmol/mg prot) (*p* < 0.05). It indicated that lipid peroxidation occurred in the HepG2 cells, induced by H_2_O_2_. After pretreatment with LMGPR, GFDGD, FGFDGDF, GHNGLDGL, and GPAGPQGPR, the MDA contents were significantly decreased to 5.91 ± 0.27 nmol/mg prot, 5.55 ± 0.16 nmol/mg prot, 3.945 ± 0.21 nmol/mg prot, 4.65 ± 0.39 nmol/mg prot, and 5.96 ± 0.30 nmol/mg prot, respectively (*p* < 0.05).

In addition, IL-8 is a pro-inflammatory cytokine, and H_2_O_2_-induced oxidative stress can trigger a cellular inflammatory response [50]. After the induction of 1.4 mM H_2_O_2_, the IL-8 content in cells significantly increased to 154.02 ± 2.80 pg/mL (*p* < 0.05), compared with that in the control group (110.92 ± 2.52 pg/mL) (Figure 8D). Under the pretreatment of five peptides (LMGPR, GFDGD, FGFDGDF, GHNGLDGL, GPAGPQGPR), the intracellular IL-8 content significantly reduced (*p* < 0.05), which indicated potential anti-inflammatory and antioxidant effects.

In general, the five peptides exhibited good cellular antioxidant activities by up-regulating the activity of intracellular antioxidant enzymes in reaction to hydrogen peroxide-induced oxidative stress and decreasing the levels of MDA and IL-8, especially FGFDGDF. The antioxidant peptides screened from YS could reduce oxidative damage and inflammation, further exerting a certain protective effect on cells. However, in vitro cell culture is usually carried out in a highly controlled environment, lacking a complex physiological environment in the body, and cannot accurately simulate the absorption and distribution of drugs in the body. So, further animal experiments and cellular markers needed to be conducted to validate the antioxidant effects and specific mechanism of these synthetic antioxidant peptides.

## 3. Materials and Methods

### 3.1. Materials and Reagents

Yak (Maiwa Yak) skins were collected and supplied from Plateau Song Food Co. Ltd. in Hongyuan County, Aba Prefecture, Sichuan Province of China, and transported to the laboratory through refrigeration under 20 °C. Trypsin (100,000 U/g) was purchased from Nanning Donghenghuadao Biotechnology Co., Ltd. (Guangxi, China). 1,1-diphenyl-2-trinitrophenylhydrazine (DPPH) was purchased from Sigma-Aldrich Chemical Co. (St. Louis, MO, USA). 2,2′-diazo-bis (3-ethylbenzothiazolin-6-sulfonic acid) diammonium salt (ABTS) was purchased from Keyuan Biochemical Co., Ltd. (Jinan, China). The HepG2 cell was donated by the laboratory of Tianjin University of Science and Technology (Tianjin, China). DMEM medium, fetal bovine serum (FBS), cell counting kit-8 (CCK-8), penicillin, and streptomycin were purchased from Dalian Meilun Biotechnology Co., Ltd. (Dalian, China). Phosphate-buffer saline (PBS) was purchased from Beijing Lanjieke Biotechnology Co., Ltd. (Beijing, China). The superoxide dismutase (SOD) test kit, hydrogen peroxide (CAT) test kit, malondialdehyde (MDA) test kit, and human interleukin 8 (IL-8) enzyme-linked immunosorbent test kits were all purchased from Nanjing Jiancheng Co., Ltd. (Nanjing, China). All other chemicals used were analytical grade.

### 3.2. Preparation of YSP by Enzymatic Hydrolysis

Enzymatic hydrolysis was adopted for the preparation of yak skin peptides (YSP). After being thawed at 4 °C, the yak skin (YS) was removed from the hair and subcutaneous connective tissue and cut into small pieces. After homogenization with water, YS powder was prepared under 80 °C, drying for 3 h and then sifted through 60 mesh. Then the YS was hydrolyzed at a ratio of powder-to-water of 1:30 (g/mL) using trypsin at a concentration of 7200 U/g in a water bath at 54 °C for 3.2 h. Subsequently, the enzyme was deactivated under 100 °C for 10 min and the supernatant was collected by centrifugation at 3500 r/min for 20 min. After being freeze-dried, the YSP was obtained and stored at −20 °C for use.

### 3.3. Fourier Transform Infrared Spectroscopy (FTIR) Analysis

The secondary structure of YS and YSP was analyzed by Fourier transform infrared spectroscopy (FTIR) with some modifications according to the previous scheme [51]. The transparent film was prepared by mixing a 1 mg sample with 150 mg potassium bromide and pressing the tablet. Then the infrared spectrum of the sample was recorded by 32 scans at a resolution of cm^−1^ in the range of 4000–500 cm^−1^. The data was analyzed using OMNIC 8.2 software.

### 3.4. Scanning Electron Microscopy Analysis

Solid samples of YS and YSP were fixed on the slide with conductive adhesive. After being coated with gold, the samples were observed using an emission scanning electron microscope at a magnification of 1000× and an acceleration voltage of 20 kV.

### 3.5. Ultrafiltration Separation

The YSPs were separated by ultrafiltration membranes with molecular weight cutoffs of 1 kDa, 3 kDa, and 10 kDa (TG 16.6, Lu Xiangyi Centrifuge Instrument Co., Ltd., Shanghai, China). Four fractions with different molecular weight were obtained, including YSP-I (<1 kDa), YSP-II (1–3 kDa), YSP-III (3–10 kDa), and YSP-IV (>1 kDa). Each fraction was gathered separately and freeze-dried for further analysis (MODULYOD-230, Thermo Fisher Technology Co. Ltd., Waltham, MA, USA).

### 3.6. Evaluation on the Free Radical Scavenging Activity

#### 3.6.1. DPPH Radical Scavenging Activity

Slightly modified according to the previous method [52], the 100 μL sample solution was mixed with 100 μL 0.1 mmol/L DPPH anhydrous ethanol solution and reacted in the dark at room temperature for 30 min. Then the absorbance was measured at 517 nm. The DPPH free radical scavenging rate of the sample was calculated by the formula below.

(1) $$DPPH\;radical\;scavenging\;rate(\%)=(1-\frac{A_{1}-A_{2}}{A_{0}})\times 100,$$

where A1 represented the absorbance value the sample reaction with DPPH anhydrous ethanol solution; A2 represented the absorbance value of the sample’s reaction with anhydrous ethanol solution; A0 represented the absorbance of the reaction mixture solution without the sample.

#### 3.6.2. ABTS Radical Scavenging Activity

Slightly modified according to the previous method [53], 7.4 mmol/L ABTS solution was mixed with the 2.6 mmol/L K_2_S_2_O_8_ solution in equal volume, and then was kept in the dark for 16 h. The ABTS working solution was obtained when the mixture was subsequently diluted with anhydrous ethanol to adjust the absorbance to a range of 0.70 ± 0.02 at 734 nm. Then, the sample solution of 40 μL was mixed with 160 μL ABTS working solution and reacted at room temperature for 10 min in the dark on 96 microporous plates. Meanwhile, the sample was replaced with distilled water as the blank. The absorbance was measured at 734 nm, and the ABTS radical scavenging rate of samples was calculated using the formula below.

(2) $$ABTS\;radical\;scavenging\;rate(\%)=\frac{A_{0}-A_{1}}{A_{1}}\times 100,$$

where *A*_0_ and *A*_1_ represented the absorbance values of the blank and sample groups.

#### 3.6.3. Hydroxyl Radical Scavenging Activity

The hydroxyl radical scavenging activities of samples were performed as in the previous method with slight modifications [54]. Briefly, 6.0 mmol/L salicylic acid solution and FeSO_4_ solution were prepared, and 0.5 mL of each solution was added into a reaction tube, followed by the addition of a 0.5 mL sample solution and a 0.5 mL H_2_O_2_ solution of 6.0 mmol/L. After mixing, the reaction was conducted in a 37 °C water bath for 0.5 h, and then the absorbance was measured at 510 nm. Meanwhile, the sample was replaced with distilled water and used as the blank group, and H_2_O_2_ was replaced with distilled water and used as the control group. The hydroxyl radical scavenging rate of the sample was calculated according to the formula below.

(3) $$hydroxyl\;radical\;scavenging\;rate(\%)=(1-\frac{A_{0}-A_{1}}{A})\times 100,$$

where *A*_0_, *A*_1,_ and *A* represented the absorbance values of the sample group, blank group, and control group, respectively.

### 3.7. Sequence Identification of YSP

The amino acid sequence of an ultrafiltration fraction with relatively high antioxidant activity was identified by LC-MS/MS. The samples were uploaded by an autosampler to Zorbax 300SB-C18 peptide traps (Agilent Technologies, Wilmington, DE, USA) and separated on a liquid chromatography column (0.15 mm × 150 mm, RP-C18, Column Technology luc., Lombard, IL, USA). The mobile phases consisted of phase A (water with 0.1% formic acid) and phase B (84% acetonitrile in water with 0.1% formic acid). The elution gradient of phase B was increased linearly from 4% to 50% in 0–50 min, increased from 50% to 100% in 50–54 min, and then kept at 100% in 54–60 min. Subsequently, the samples and their fragments were analyzed using a Q Exactive HF-X mass spectrometer (Thermo Fisher, USA) with a positive ion for 60 min. The m/z of peptides and their fragments were collected according to the 10 fragmentation profiles after each full scan. The amino acid sequences of peptides were identified and quantified by searching the corresponding database with MaxQuant 1.5.5.1.

### 3.8. Online Informatics Screening of Potential Antioxidant Peptides

Peptide sequences with high activity, non-toxicity, and good water solubility were screened. The Peptide Ranker (http://distilldeep.ucd.ie/PeptideRanker/) (accessed on 18 November 2023) and ToxinPred (http://crdd.osdd.net/raghava/toxinpred/index.html) (accessed on 18 November 2023) online programs were used to predict peptide bioactivity probability and the toxicity of the peptides [55,56]. In addition, the water solubility of peptides was also determined using the online computational tool Innovagen (http://www.innovagen.com/proteomics-tools) (accessed on 18 November 2023) [31].

### 3.9. Molecular Docking of YSP with Keap1 Protein

The interactions between peptides and Keap1 protein were investigated by molecular docking. Firstly, the 2D structural formula of peptides were drawn using Kingdraw Offical 4.0 software, and transformed into 3D structures by Kingdraw Offical 4.0 software. MM2 in the calculation function was selected for the energy minimization of peptides, then, the small ligand molecules of peptides were obtained. The crystal structure of the Keap1-Nrf2 complex (PDB ID: 2FLU) was obtained from the PDB protein database. Afterwards, the molecular docking was performed using Autodock 4.2.3. Water molecules, small solvent molecules, Nrf2 16-mer peptides, and other ligands were removed from the protein structure, and the protein was hydrogenated. The molecular docking mode was performed based on semi-flexible molecular docking (CDOCKER), with the receptor protein set to rigid and the ligand small molecule set to flexible [20]. The active pocket center coordinates were x: 5, y: 9, and z: 1, and the radius was 15 Å. The CDOCKER_ENERGY value, interaction sites, and intermolecular action forces were used to analyze the molecular docking results. The 3D and 2D interactions between the peptides and Keap1 were visualized using PyMol 2.5 software.

### 3.10. Synthesis of Antioxidant Peptides

The screened peptides with potential antioxidant activity were further synthesized with purity over 90%, as in the solid phase method explored by Nanjing Yuan-Peptide Biotechnology Co., Ltd. (Nanjing, China). Then the free radical scavenging activities and cellular antioxidant activities of the synthesized peptides were evaluated.

### 3.11. Evaluation on the Cellular Antioxidant Activity of YSP

#### 3.11.1. Cell Culture

HepG2 cells were cultured in DMEM growth medium supplemented with 10% FBS and 50 U/mL penicillin-streptomycin in a culture flask. The flask was incubated in an incubator of 5% CO_2_ and kept at 37 °C. The culture medium was refreshed every 2–3 days. Once the HepG2 cells adhered to the flask wall and achieved a coverage rate of over 85%, the cells were digested using a 0.25% trypsin solution for passage.

#### 3.11.2. Determination of Cell Viability

Cell viability was determined by the CCK-8 method. The synthesized peptides were dissolved in DMEM growth medium containing 10% FBS with concentrations of 0.1, 0.2, 0.5, 1.0, and 2.5 mg/mL. HepG2 cell suspensions were seeded in 96-well plates at a density of 5 × 10^4^ cells/mL with 200 μL in each well, and cultured for 24 h until the cell fusion reached about 85%. After washing twice with PBS, 100 μL of each peptide solution was added to the cells and incubated for 24 h. Finally, 90 μL of culture medium along with 10 μL of CCK-8 reagent were added to each well for a further 2 h incubation. Then the absorbance of each well was measured at 450 nm using a microplate reader. Meanwhile, a blank group without cells and a control group without peptides were established. The cell viability was calculated using the formula below.

(4) $$\mathrm{Cell}\;\mathrm{viability}=(\frac{As-A_{0}}{Ac-A_{0}})\times 100$$

*As* represented the absorbance of sample treatment, while *A*_0_ and *Ac* represented the absorbances of the blank and control groups.

#### 3.11.3. Establishment of Oxidative Stress Model in HepG2 Cells

HepG2 cells were cultured in 96-well plates at a density of 5 × 10^4^ cells/mL for 24 h. After the medium was removed, 100 µL of complete medium containing different concentrations of H_2_O_2_ (800, 1000, 1200, 1400, 1600 µM) was added and incubated for 2 h. After the medium was discarded, cell viability was determined using the CCK-8 assay to establish the optimal H_2_O_2_ concentration for inducing an oxidative stress model. The cells without H_2_O_2_ treatment were used as control group.

#### 3.11.4. Treatment of Peptides on H_2_O_2_-Induced HepG2 Cells

HepG2 cells were cultured in 96-well plates at a density of 5 × 10^4^ cells/mL for 24 h until the cell confluence reached about 85%. After the medium was discarded, 100 μL peptides of different concentrations were added to the wells and cultured for an additional 24 h. After discarding the medium and then being washed, the cells were incubated with 100 μL of an appropriate concentration of H_2_O_2_. After incubation for 2 h, the supernatant was collected, and the content of IL-8 was determined strictly, according to the kit instructions. Afterwards, the cells were washed twice with PBS, and then the cell viability was measured. Alternatively, the cells were disrupted with 300 μL of cell lysate and centrifuged at 6000× *g* at 4 °C for 15 min. The supernatant was collected and stored at −80 °C. The protein concentration was determined using the Bio-Rad DC analysis with BSA as the standard curve. In addition, the contents of SOD, CAT, and MDA in cell lysates were determined in strict accordance with the kit instructions.

### 3.12. Statistical Analysis

All experiments were repeated at least three times, and the data were expressed as mean ± standard deviation. Prism 9 and PyMol 2.5 were used for statistical analysis and data plotting. One way ANOVA was performed for significance difference analysis and multiple comparison, where *p* < 0.05 represented a significant difference between the data.

## 4. Conclusions

In conclusion, YSP was prepared and analyzed using the LC-MS/MS technique in this study. A total of 219 peptides were identified and five peptides with high scores (LMGPR, GFDGD, FGFDGDF, GHNGLDGL, and GPAGPQGPR) were selected for verification through molecular docking prediction with Keap1. Through a H_2_O_2_-induced oxidative HepG2 cell model, these five antioxidant peptides demonstrated a significant protective effect by increasing the activity of intracellular antioxidant enzymes and reducing the levels of MDA and IL-8. Among them, FGFDGDF exhibited relatively strong free radical scavenging activities and protective effects. These findings provide support for the application of YSP as a new antioxidant in functional foods or in the treatment of diseases related to oxidative stress. Future research may explore the application of YSP as a food additive to enhance the taste, color, and nutritional value of food products. In the cosmetics industry, YSP could serve as an active ingredient to augment the moisturizing, sunscreen, and antioxidant properties of cosmetic formulations.

## Figures and Tables

**Table 1:** A total of 29 antioxidant peptides screened with good water solubility and non-toxicity and their binding energy of interaction with Keap1.

NO.	Sequence	Length	Mass	Score	Peptide Ranker	CDockerenergy (kcal/mol)
1	FGFDGDF	7	803.31262	187.48	0.93535	−141.445
2	GHNGLDGL	8	781.37187	114.54	0.534861	−136.723
3	GFDGDF	6	656.24421	148.12	0.913215	−119.541
4	FDGDF	5	599.22274	78.156	0.94884	−116.697
5	GFDGD	5	509.17579	118.96	0.637708	−115.582
6	GPSGPPGPDGN	11	950.40937	206.38	0.839787	−90.731
7	FEL	3	407.20564	18.31	0.566207	−84.218
8	GPAGPQGPR	9	835.43005	183.43	0.846699	−83.766
9	GFE	3	351.14304	12.647	0.720069	−83.496
10	GPAGPAGRPG	10	835.43005	131.04	0.873123	−82.544
11	FGE	3	351.14304	65.815	0.675338	−82.025
12	GEGGPQGPR	9	853.40423	142.64	0.536924	−81.513
13	WDT	3	420.1645	25.115	0.593031	−81.295
14	DML	3	377.16206	25.115	0.667515	−80.952
15	MDL	3	377.16206	47.364	0.679551	−78.408
16	WAD	3	390.15393	20.412	0.721933	−77.465
17	GPAGPAGRP	9	778.40859	155.73	0.846699	−71.902
18	PGPAGPAGRP	10	875.46135	107.32	0.871669	−71.298
19	DWP	3	416.16958	20.675	0.948036	−69.084
20	LMGPR	5	572.31045	85.518	0.767987	−68.852
21	PAGPAGPR	8	721.38712	109.7	0.813961	−68.468
22	DPC	3	333.09946	5.9869	0.819965	−68.046
23	FSGLD	5	537.24348	41.717	0.692962	−67.018
24	FCK	3	396.18313	11.715	0.907916	−62.174
25	GPK	3	300.17976	40.566	0.567438	−45.198
26	PQPPQ	5	565.28601	104.03	0.631168	−42.868
27	PAGRP	5	496.27578	91.004	0.74837	−42.160
28	PGS	3	259.11682	11.355	0.526816	−38.120
29	PGR	3	328.1859	32.689	0.839926	−34.945

## Data Availability

The data that support the findings of this study are available from the first author upon reasonable request.

## Supplementary Materials

The following supporting information can be downloaded at: https://www.mdpi.com/article/10.3390/ijms26135976/s1.
