# Supplementary material for: Identification and Screening of Novel Antioxidant Peptides from Yak Skin and Their Protective Effect on H2O2-Induced HepG2 Cells Oxidation
Source: Int J Mol Sci. 2025 Jun 21;26(13):5976. doi: 10.3390/ijms26135976 (PMC12249663; doi:10.3390/ijms26135976)
Supplement: Supplementary file 1 [file ijms-26-05976-s001.zip › ijms-3668269-SI.pdf]

Supplementary Table S1. The 219 antioxidant peptide sequences identified by LC-MS/MS technology

NO.	Sequence	Length	Mass	Charge	Score	Peptide ranker
1	PGA	3	243.12191	1	9.64E-16	0.674335
2	SNA	3	290.12263	1	9.64E-16	0.121905
3	VVC	3	319.15658	1	9.64E-16	0.130272
4	VRG	3	330.20155	1	9.64E-16	0.272598
5	PFA	3	333.16886	1	9.64E-16	0.925891
6	TQP	3	344.16958	2	9.64E-16	0.168096
7	VVE	3	345.18999	3	9.64E-16	0.0218372
8	SRS	3	348.17573	3	9.64E-16	0.185601
9	VHC	3	357.14707	3	9.64E-16	0.28233
10	PNK	3	357.20122	1	9.64E-16	0.230513
11	NKC	3	363.15764	3	9.64E-16	0.317728
12	YLA	3	365.19507	1	9.64E-16	0.33866
13	MLL	3	375.21918	1	9.64E-16	0.820396
14	WVS	3	390.19032	1	9.64E-16	0.397029
15	VFQ	3	392.20597	1	9.64E-16	0.39097
16	QMK	3	405.20459	3	9.64E-16	0.317534
17	PFF	3	409.20016	1	9.64E-16	0.996755
18	QKH	3	411.22302	2	9.64E-16	0.0988496
19	MKH	3	414.20492	1	9.64E-16	0.331692
20	YTH	3	419.18048	1	9.64E-16	0.134949
21	YHC	3	421.14199	2	9.64E-16	0.64542
22	YSR	3	424.20703	1	9.64E-16	0.317883
23	YLH	3	431.21687	1	9.64E-16	0.394538
24	QFF	3	440.20597	3	9.64E-16	0.990589
25	WHC	3	444.15797	3	9.64E-16	0.964383
26	WKL	3	445.2689	1	9.64E-16	0.827822
27	WLE	3	446.21653	1	9.64E-16	0.592565
28	YKH	3	446.22777	1	9.64E-16	0.167029
29	YLY	3	457.22129	1	9.64E-16	0.53811
30	WQK	3	460.24342	1	9.64E-16	0.573435
31	WQE	3	461.19105	1	9.64E-16	0.362335
32	WLF	3	464.24236	1	9.64E-16	0.995262
33	WKH	3	469.24375	1	9.64E-16	0.658682
34	WRE	3	489.23358	2	9.64E-16	0.623858
35	PGK	3	300.17976	2	4.34E-15	0.517877
36	VPS	3	301.16377	1	4.34E-15	0.153453
37	DSP	3	317.1223	1	4.34E-15	0.317559
38	SPD	3	317.1223	1	4.34E-15	0.348427

39	HAR	3	382.2077	1	4.34E-15	0.264117
40	WAE	3	404.16958	1	4.34E-15	0.43189
41	LFE	3	407.20564	1	4.34E-15	0.508748
42	RRN	3	444.25571	1	4.34E-15	0.349795
43	GEM	3	335.11511	1	3.2581	0.405945
44	VSM	3	335.15149	1	4.2485	0.211523
45	FEN	3	408.1645	1	4.2878	0.323703
46	VPG	3	271.15321	2	4.6357	0.430505
47	EPC	3	347.11511	1	4.6357	0.45965
48	YPGR	4	491.24923	2	5.2048	0.799204
49	SPL	3	315.17942	1	5.7496	0.665423
50	TLC	3	335.15149	1	5.7496	0.421461
51	TTM	3	351.14641	1	5.7496	0.137765
52	DPC	3	333.09946	1	5.9869	0.819965
53	RGPG	4	385.20737	2	6.0458	0.780033
54	LVAK	4	429.29512	2	6.0458	0.07706
55	LERP	4	513.2911	2	6.0458	0.236101
56	LRML	4	531.32029	2	6.0458	0.745298
57	TNW	3	419.18048	1	6.6306	0.55469
58	EESP	4	460.18054	2	6.6306	0.069204
59	QDCV	4	463.17368	2	6.6306	0.272948
60	WGS	3	348.14337	1	7.337	0.873369
61	LSW	3	404.20597	1	7.337	0.82823
62	AAD	3	275.11174	1	8.2202	0.137693
63	PFG	3	319.15321	1	8.2202	0.982325
64	YPG	3	335.14812	1	8.2202	0.825303
65	LLL	3	357.26276	1	8.2202	0.568242
66	FVP	3	361.20016	1	8.2202	0.827943
67	LLM	3	375.21918	1	8.2202	0.775378
68	VEF	3	393.18999	1	8.2202	0.218709
69	YSK	3	396.20088	1	8.2202	0.131201
70	LFM	3	409.20353	1	8.2202	0.980285
71	WVM	3	434.19878	1	8.2202	0.901786
72	WPH	3	438.20155	2	8.2202	0.954735
73	FFF	3	459.21581	1	8.2202	0.99883
74	WWP	3	487.22195	1	8.2202	0.996803
75	LVAH	4	438.25907	2	8.7137	0.107156
76	RFVP	4	517.30127	2	9.0142	0.690733
77	DKP	3	358.18523	1	10.154	0.252231
78	LEV	3	359.20564	1	10.891	0.0386995
79	AGRP	4	399.22302	2	11.015	0.748362
80	PGS	3	259.11682	1	11.355	0.526816
81	SMP	3	333.13584	1	11.715	0.802974
82	FCK	3	396.18313	1	11.715	0.907916

83	LPV	3	327.21581	1	12.049	0.296867
84	WLA	3	388.21106	1	12.049	0.888602
85	GFE	3	351.14304	1	12.647	0.720069
86	TSY	3	369.1536	1	12.647	0.100025
87	WSE	3	420.1645	1	13.38	0.36701
88	WPG	3	358.16411	1	13.669	0.986609
89	WSG	3	348.14337	1	13.717	0.903678
90	LEL	3	373.22129	1	13.717	0.114531
91	WTV	3	404.20597	1	14.966	0.310686
92	LRTL	4	501.32748	2	15.259	0.292505
93	YSRP	4	521.2598	2	15.319	0.559207
94	LRPL	4	497.33257	2	15.393	0.707513
95	YFP	3	425.19507	1	15.73	0.972428
96	LFT	3	379.21072	1	16.46	0.717909
97	VPLR	4	483.31692	2	16.757	0.404016
98	PGE	3	301.12739	1	16.863	0.324786
99	LNL	3	358.22162	1	16.863	0.324786
100	FPM	3	393.17223	1	17.009	0.991805
101	TGP	3	273.13247	1	18.31	0.460722
102	TPA	3	287.14812	1	18.31	0.226061
103	VEV	3	345.18999	1	18.31	0.0213037
104	SGW	3	348.14337	1	18.31	0.943192
105	PYQ	3	406.18523	1	18.31	0.433994
106	FEL	3	407.20564	1	18.31	0.566207
107	SWE	3	420.1645	1	18.31	0.467259
108	WRP	3	457.24375	2	18.31	0.977529
109	MRW	3	491.23147	2	18.31	0.978884
110	PGL	3	285.16886	1	20.412	0.855192
111	LFP	3	375.21581	1	20.412	0.971355
112	WAD	3	390.15393	1	20.412	0.721933
113	DWP	3	416.16958	1	20.675	0.948036
114	PGKP	4	397.23252	2	21.483	0.672456
115	LPR	3	384.2485	2	22.337	0.694502
116	PVP	3	311.18451	1	23.112	0.488562
117	WSP	3	388.17467	1	23.444	0.931165
118	LLEK	4	501.31625	2	23.77	0.0835857
119	LRF	3	434.26415	2	24.243	0.951036
120	FPG	3	319.15321	1	24.318	0.985903
121	DML	3	377.16206	1	25.115	0.667515
122	WDT	3	420.1645	1	25.115	0.593031
123	QLP	3	356.20597	1	27.055	0.562817
124	VPF	3	361.20016	1	27.155	0.873193
125	LWG	3	374.19541	1	28.861	0.961165
126	LSM	3	349.16714	1	28.962	0.513743

127	LLAH	4	452.27472	2	30.717	0.239269
128	EWV	3	432.20088	1	30.835	0.227203
129	FPS	3	349.16377	1	32.202	0.905231
130	LRLE	4	529.3224	2	32.323	0.134714
131	PGR	3	328.1859	1	32.689	0.839926
132	GPA	3	243.12191	1	33.176	0.725277
133	VGF	3	321.16886	1	33.176	0.829794
134	YPV	3	377.19507	1	33.176	0.327902
135	QEW	3	461.19105	1	34.935	0.43769
136	LHPL	4	478.29037	2	34.935	0.593845
137	FPA	3	333.16886	1	36.05	0.951498
138	PGP	3	269.13756	1	36.833	0.908686
139	GPY	3	335.14812	1	36.833	0.85884
140	WVP	3	400.21106	1;2	36.833	0.865855
141	SLM	3	349.16714	1	37.174	0.679551
142	FAP	3	333.16886	1	38.344	0.944959
143	GPK	3	300.17976	1	40.566	0.567438
144	VFT	3	365.19507	1	40.635	0.340223
145	FSGLD	5	537.24348	1	41.717	0.692962
146	GFPGA	5	447.21178	1	41.719	0.891436
147	FPGAP	5	487.24308	1	41.719	0.948409
148	GLV	3	287.18451	1	41.83	0.277867
149	SFP	3	349.16377	1	42.098	0.939625
150	TLM	3	363.18279	1	42.098	0.428067
151	TYVPK	5	606.33771	2	42.565	0.117894
152	GPR	3	328.1859	1;2	42.596	0.865974
153	FPN	3	376.17467	1	44.439	0.939417
154	QGLPG	5	470.2489	1	45.108	0.615998
155	LTGPL	5	499.3006	1	46.402	0.501266
156	MDL	3	377.16206	1	47.364	0.679551
157	LSY	3	381.18999	1	47.364	0.24612
158	GPMGPR	6	613.30062	2	47.403	0.891703
159	SLL	3	331.21072	1	49.069	0.451826
160	YPL	3	391.21072	1	52.041	0.792883
161	GPSGP	5	413.19105	1	52.958	0.730188
162	VLP	3	327.21581	1	55.918	0.330829
163	LGAGL	5	429.25873	1	58.511	0.517362
164	SWP	3	388.17467	1	61.092	0.949184
165	LLEPG	5	527.29551	1	61.26	0.292154
166	LLT	3	345.22637	1	62.263	0.220663
167	FGE	3	351.14304	1	65.815	0.675338
168	VGAPGPK	7	624.35951	2	65.897	0.482923
169	PGAPG	5	397.19613	1	72.087	0.699841
170	LGPVG	5	441.25873	1	72.087	0.478309

171	GGPQGPR	7	667.34017	2	74.832	0.780663
172	LGGLG	5	415.24308	1	76.733	0.616854
173	GFAGP	5	447.21178	1	76.733	0.832118
174	PGPMG	5	457.1995	1	76.733	0.926465
175	PAGPR	5	496.27578	2	77.925	0.733205
176	PMGPR	5	556.27915	2	77.925	0.872946
177	FDGDF	5	599.22274	1	78.156	0.94884
178	LGMGPR	6	629.33192	2	81.297	0.726598
179	FAGPA	5	461.22743	1	82.235	0.821762
180	LMGPR	5	572.31045	2	85.518	0.767987
181	APGLP	5	453.25873	1	87.753	0.832799
182	PAGRP	5	496.27578	2	91.004	0.74837
183	GLTGL	5	459.2693	1	99.626	0.422424
184	PTGPL	5	483.2693	1	102.87	0.666024
185	GPPGPAG	7	551.27036	1	102.87	0.823583
186	PQPPQ	5	565.28601	1	104.03	0.631168
187	GLMGP	5	473.2308	1	104.08	0.815314
188	FPGAV	5	489.25873	1	104.08	0.765628
189	GPPGNVGNPGV	11	963.47739	2	104.45	0.643855
190	GFSGL	5	479.238	1	105.36	0.844211
191	PGPAGPAGRP	10	875.46135	2	107.32	0.871669
192	LSPIFPGGA	9	857.4647	1	107.43	0.830495
193	PAGPAGPR	8	721.38712	2	109.7	0.813961
194	PGAVGPAGPR	10	877.477	2	111.04	0.659018
195	PGPAGPR	7	650.35001	2	112.27	0.845188
196	GHNGLDGL	8	781.37187	2	114.54	0.534861
197	LPQPP	5	550.3115	1	116.54	0.765628
198	GPQGPR	6	610.31871	2	118.18	0.783012
199	GFDGD	5	509.17579	1	118.96	0.637708
200	KIVVHP	6	691.4381	2	119.58	0.105779
201	GPAGPAGRPG	10	835.43005	2	131.04	0.873123
202	GPVGPVG	7	581.31731	1	132.53	0.519258
203	VGPAGPNG	8	667.32894	1	134.12	0.572237
204	ALVPGGPA	8	680.38573	1	136.94	0.371191
205	GPPGPIGN	8	707.36024	1	141.55	0.832069
206	GEGGPQGPR	9	853.40423	2	142.64	0.536924
207	LPQPPQ	6	678.37008	1	146.24	0.570392
208	GPAGPPGPIGN	11	932.47158	1;2	147.69	0.910197
209	GFDGDF	6	656.24421	1	148.12	0.913215
210	SGAAGPTGPIGS	12	970.47197	2	151.97	0.564037
211	GFPGLPGPS	9	827.41775	1	153.26	0.775476
212	GPAGPAGRP	9	778.40859	2	155.73	0.846699
213	GPAGPIGPV	9	763.42284	1	161.77	0.761899
214	GLTGPL	6	556.32206	1	164.54	0.585058

215	LKPDPNT	7	783.41267	2	165.52	0.252501
216	AGPSGPAGPTG	11	867.40865	1	182.53	0.722641
217	GPAGPQGPR	9	835.43005	2	183.43	0.846699
218	FGFDGDF	7	803.31262	1	187.48	0.93535
219	GPSGPPGPDGN	11	950.40937	2	206.38	0.839787
